# Serological testing for Hansen’s disease diagnosis: Clinical significance and performance of IgA, IgM, and IgG antibodies against Mce1A protein

**DOI:** 10.3389/fmed.2023.1048759

**Published:** 2023-03-16

**Authors:** Filipe Rocha Lima, Mateus Mendonça Ramos Simões, Gabriel Martins da Costa Manso, Diana Mota Toro, Vanderson Mayron Granemann Antunes, Giovani Cesar Felisbino, Gabriela Ferreira Dias, Lee W. Riley, Sérgio Arruda, Natália Aparecida de Paula, Helena Barbosa Lugão, Fernanda André Martins Cruz Perecin, Norma Tiraboschi Foss, Marco Andrey Cipriani Frade

**Affiliations:** ^1^Healing and Hansen’s Disease Laboratory, Ribeirão Preto Medical School, University of São Paulo, São Paulo, Brazil; ^2^Dermatology Division, Department of Internal Medicine, National Referral Center for Sanitary Dermatology and Hansen’s Disease, University Hospital, Ribeirão Preto Medical School, University of São Paulo, São Paulo, Brazil; ^3^Department of Clinical, Toxicological and, Bromatological Analyses, School of Pharmaceutical Sciences of Ribeirão Preto, University of São Paulo, São Paulo, Brazil; ^4^Division of Infectious Diseases and Vaccinology, School of Public Health, University of California, Berkeley, Berkeley, CA, United States; ^5^Advanced Public Health Laboratory, Gonçalo Moniz Institute, Oswaldo Cruz Foundation, Salvador, Brazil

**Keywords:** Hansen’s disease, testing, serological, diagnosis, Mce1A, antibodies

## Abstract

Hansen’s disease (HD) is an infectious, treatable, and chronic disease. It is the main cause of infectious peripheral neuropathy. Due to the current limitations of laboratory tests for the diagnosis of HD, early identification of infected contacts is an important factor that would allow us to control the magnitude of this disease in terms of world public health. Thus, a cross-sectional study was conducted in the Brazilian southeast with the objective of evaluating humoral immunity and describing the accuracy of the immunoassay based on IgA, IgM, and IgG antibodies against surface protein Mce1A of *Mycobacterium*, the predictive potential of these molecules, the clinical significance of positivity, and the ability to segregate new HD cases (NC; *n* = 200), contacts (HHC; *n* = 105), and healthy endemic controls (HEC; *n* = 100) as compared to α-PGL-I serology. α-Mce1A levels for all tested antibodies were significantly higher in NC and HHC than in HEC (*p* < 0.0001). The performance of the assay using IgA and IgM antibodies was rated as highly accurate (AUC > 0.85) for screening HD patients. Among HD patients (NC), positivity was 77.5% for IgA α-Mce1A ELISA, 76.5% for IgM, and 61.5% for IgG, while α-PGL-I serology showed only 28.0% positivity. Multivariate PLS-DA showed two defined clusters for the HEC and NC groups [accuracy = 0.95 (SD = 0.008)] and the HEC and HHC groups [accuracy = 0.93 (SD = 0.011)]. IgA was the antibody most responsible for clustering HHC as compared to NC and HEC, evidencing its usefulness for host mucosal immunity and as an immunological marker in laboratory tests. IgM is the key antibody for the clustering of NC patients. Positive results with high antibody levels indicate priority for screening, new clinical and laboratory evaluations, and monitoring of contacts, mainly with antibody indexes ≥2.0. In light of recent developments, the incorporation of new diagnostic technologies permits to eliminate the main gaps in the laboratory diagnosis of HD, with the implementation of tools of greater sensitivity and accuracy while maintaining satisfactory specificity.

## 1. Introduction

Hansen’s disease (HD) is an infectious and contagious disease that mainly affects the skin, the peripheral nerves, mucosa of the upper respiratory tract, and the eyes, being caused by bacilli of the *Mycobacterium leprae* complex, which includes *M. leprae* and *M. lepromatosis* (1). HD is the most common treatable cause of peripheral neuropathy; however, it can progress to physical disabilities and deformities in the absence of an early diagnosis and the implementation of effective multidrug therapy (MDT) (2). HD is classified as a major public health issue and in 2019, with more than 200,000 new cases of HD reported worldwide and 27,864 reported in Brazil, a value equivalent to 93% of all cases in the Americas region and to 13.7% of the global cases registered. The heterogeneous distribution and the epidemiological indicators of Brazil at the global level reveal a scenario of continued transmission, with the disease representing a priority among the health problems of the country (3). According to the World Health Organization (WHO), as a result of the impact of the COVID-19 pandemic, more than 120,000 new cases were reported in 2020, with a 37% reduction compared to 2019 (4).

The incorporation of new laboratory technologies for an early diagnosis of HD and the identification of infected individuals will allow the control of the transmission chain and the global magnitude of the disease, as proposed by the WHO strategies (3). Thus, the absence of high performance diagnostic platforms for the diagnosis of patients across the clinical spectrum of the disease and of oligosymptomatic household contacts (HHC) are gaps in health units that do not allow early case detection, accurate diagnosis, or prompt treatment. Currently, anti-phenolic glycolipid-I (α-PGL-I) serology is the most widespread tool for the complementary diagnosis of the disease and contact with *M. leprae* based on antibody research. However, due to the low and variable sensitivity and negative predictive value of this test, as well as its low ability to detect early cases, paucibacillary patients, and macular and neural forms, its accuracy is not satisfactory for use as a diagnostic laboratory tool (5–7). Parallel to this, the slit-skin smear and the anatomopathological examination of the skin biopsy, despite having high specificity, are also techniques that depend on the bacillary load of the host and are of low sensitivity for effective detection and screening of HD cases and their HHC (6, 7). More recently, the introduction of molecular biology to identify bacillus DNA in clinical samples (skin, nasal swab, and intradermal scraping) has increased the probability of detecting new cases while maintaining high specificity and has shown that the polymerase chain reaction (PCR) may be used to confirm most field cases (8). On the other hand, PCR is an expensive method not available to all laboratories for the diagnosis of HD, in addition to the absence of a gold standard laboratory test (7).

To validate new biomarkers for the diagnosis of all clinical forms of HD, infected individuals, and characterization of these molecules in the population residing in an endemic region, antibodies against the mammalian cell-entry protein 1A (Mce1A) of *Mycobacterium* were evaluated. Mce1A is reported to mediate bacillus entry into cells in the host’s reticuloendothelial system cells and to induce their survival (9, 10). Despite the presence of the Mce1A protein in the *Mycobacterium* genus, preliminary studies have shown that conditions such as bacillus Calmette-Guérin (BCG) vaccination and latent tuberculosis infection (LTBI) do not interfere with the levels of anti-Mce1A antibodies (α-Mce1A) in HD patients (11, 12). Previously published studies have reported the potential of α-Mce1A antibodies for the detection and monitoring of HD, also indicating its role in the identification of asymptomatic contacts (11, 12). However, the present study is the first one carried out in a state of low endemicity in the Brazilian southeast, including patients with macular forms and mainly neurological signs and symptoms, representing the largest sample tested for the proposed serological assay. Thus, determining the most appropriate test cut-off value for each region and each biomarker. On this basis, our study aimed to describe the accuracy of an immunoassay based on α-Mce1A IgA, IgM, and IgG antibodies, as well as the predictive potential of these molecules, the clinical significance of positivity and their ability to segregate HD patients, contacts, and healthy endemic controls.

## 2. Materials and methods

### 2.1. Design and study population

A cross-sectional study was conducted at the National Referral Center in Sanitary Dermatology and HD, University Hospital of the Ribeirão Preto Medical School (HCFMRP-USP), University of São Paulo, Brazil, from 2020 to 2022. The study population (*N* = 405) was classified into three groups: new HD cases without MDT (NC), household contacts of HD patients (HHC), and healthy endemic controls (HEC).

#### 2.1.1. New HD cases (NC)

NC (*n* = 200) were diagnosed by clinical evaluation according to the Brazilian Ministry of Health and WHO guidelines using recommended cardinal signs (13). The dermatological and neurological evaluation of the patients was the confirmatory exam performed by dermatologists and leprologists for the diagnosis of HD. Auxiliary tests to the clinical diagnosis were used, such as assessment of tactile sensation with a Semmes-Weinstein esthesiometer, ultrasound of peripheral nerves, and electroneuromyography, besides complementary exams such as serology, molecular exams, and bacilloscopy. Considering that none of the classifications for HD include all of the clinical manifestations of HD, particularly those involving macular and pure neural forms, we classified the patients considering the guidelines adapted by Madrid (Congress of Madrid 1953) and the Indian Association of Leprology (IAL 1982) classifications as follows: indeterminate (I), polar tuberculoid (TT), borderline (B), borderline lepromatous (BL), polar lepromatous (LL), and pure neural (N); and PB (I and TT clinical forms) and MB (B, BL, LL, and N forms) according to the WHO operational criteria. Considering the classification by Frade et al. (14), patients with atypical hypochromatic macules and with altered sensation and neurological findings were classified as having the B and MB forms. All newly diagnosed patients were referred to a health unit for standard MDT.

#### 2.1.2. Household contacts (HHC)

HHC (*n* = 105) were defined as individuals residing or having resided in the same household with an HD patient in the last 5 years at the time of diagnosis (3). All HHC were clinically screened for signs and symptoms of HD and subjected to laboratory analysis with serological and molecular exams. Clinical examinations were performed by dermatologists and leprologists at HCFMRP-USP.

#### 2.1.3. Healthy endemic controls (HEC)

HEC (*n* = 100), representing community contacts, were defined as healthy individuals residing in the Ribeirão Preto region, SP, Brazil. During the last 5 years (2018 to 2022), the state was classified as having low endemicity. The Ribeirão Preto municipality was classified as having very high endemicity in 2021 for the first time during the study period, according to the new case detection rate of the disease. All participants reported that they had no history of diagnosis or contact with an HD, were test-negative for human immunodeficiency virus (HIV), had no diseases, and did not use immunosuppressive drugs.

### 2.2. Anti-PGL-I serology

Indirect ELISA was used to measure the α-PGL-I IgM titer of every serum sample and the cut-off was based on the OD average among healthy subjects multiplied by 2.1 plus 10%, according to a previously reported protocol (7, 12, 15). Serology was performed with an ND-O-BSA (PGL-I)-based glycoconjugate of bovine serum albumin (NR-19346. BEI Resources).

### 2.3. Molecular diagnosis of *Mycobacterium leprae* DNA

Total DNA extraction from a skin biopsy and/or earlobes and at least one elbow, knee and/or lesion slit-skin smear sample was performed with the QIAamp DNA Mini Kit (Qiagen, Germantown, MD, cat: 51306) according to the manufacturer’s protocol. DNA was used to perform quantitative PCR-RLEP according to a previously reported protocol (7, 16). The quantitative PCR (qPCR) result was considered positive for the detection of *M. leprae* DNA with amplification up to a 40.0 cycle threshold (Ct) and melting temperature at 87.5°C. The maximum number of cycles used was 40.0.

### 2.4. Anti-Mce1A serological testing

Quantitative evaluation of IgA, IgM, and IgG antibody α-Mce1A protein was performed by indirect ELISA according to a previously reported protocol (7, 11, 12). Purified recombinant Mce1A protein was provided by Dr. LW Riley (University of California, Berkeley, CA, USA). The respective index was calculated by dividing the optical density (OD 450 nm) of each sample by the cut-off, with indexes above 1.0 being considered positive. The cut-off point was based on mean OD between healthy controls compared to samples from patients with HD. The OD data were analyzed by receiver operating characteristic (ROC) curves to determine the cut-off point highest and matched sensitivity, specificity, and likelihood ratio, as previously described (7, 11, 12). For all assays, negative control samples from healthy individuals with no history of diagnosis or contact with HD, positive samples for α-Mce1A antibodies from patients diagnosed with HD, and wells considered blank without the addition of specific antibodies and with peroxidase-linked second antibody for each immunoglobulin tested were added. The OD values of the blank wells were used for subtraction in the respective results obtained in each well with the tested samples.

### 2.5. Statistical analysis

Data were analyzed with GraphPad Prism v. 9.0 software (GraphPad Inc., La Jolla, CA, USA). Study population characteristics were analyzed by the t test and Chi-squared test. Antibody level variations were analyzed by the Kruskal–Wallis test, followed by Dunn’s test. The ability of immunoglobulin levels to discriminate NC and HHC from HEC was evaluated by ROC curves. The accuracy classification was based on Bowers et al. (17). The level of statistical significance was set at *p* < 0.05. The combined performance of the antibodies in distinguishing the groups was determined using Python 3.9.12 in the Jupyter Notebook environment. The libraries used were Numpy 1.21.5, Pandas 1.4.2, Matplotlib 3.5.1, Scipy 1.7.3, Sklearn 1.0.2, and Shap 0.40.0. Data were first anonymized and all patient identification was excluded from the database. For multivariate analysis, the dataset variables were transformed using the Partial Least Square method and the two latent variables that explained most of the variance were used to construct the graphs. The Mahalanobis distance and the Chi-square distribution with a threshold of 0.95 were used to detect outliers. Partial Least Square-Discriminant Analysis (PLS-DA) was implemented with a stratified cross-validation of 10 divisions and 20 repetitions and a variable importance in projection (VIP) score plot for important antibody identified by PLS-DA analysis was evaluated. The VIP score value closest to or greater than 1 is the of rule thumb for selecting relevant variables. Thus, to investigate the importance of the variables, the Shapley values of each individual for each of the antibodies were obtained and the mean of the module of these values was then calculated. Spearman’s correlation was used to compare the antibody levels and classification was based on Akoglu (18). Finally, Hierarchical clustering was performed using Euclidean distance and Ward’s linkage algorithms were performed using MetaboAnalyst 5.0. The analyzes were carried out with the antibody indexes corresponding to each group under study, and all input data have been normalized and transformed into logarithm. Two parameters were considered to perform hierarchical clustering. The first one is similarity measure—Euclidean distance, Pearson’s correlation, and Spearman’s rank correlation. The other parameter is clustering algorithms, including average linkage (clustering uses the centroids of the observations), complete linkage (clustering uses the farthest pair of observations between the two groups), single linkage (clustering uses the closest pair of observations), and Ward’s linkage (clustering to minimize the sum of squares of any two clusters). Heatmap was presented as a visual aid in addition to the dendrogram also showing distance measure using Euclidean and clustering algorithm using ward.D, where dendrogram data values are transformed to an average color scale displaying high values in red and low values in blue. The study was developed with pre-specified tests and considering α-PGL-I ELISA and PCR as reference standard and α-Mce1A ELISA as index test.

## 3. Role of the funding source

The funder of the study had no role in the study design, data collection, data analysis, data interpretation, or writing of the report. All authors had full access to all of the data in the study and had final responsibility for the decision to submit for publication.

## 4. Results

### 4.1. Clinical and demographic findings

The spontaneous demand for care at the health unit did not permit the recruitment of a population with no statistically significant difference in terms of age, which on average ranged from 41.1 to 58.5 years (*p* < 0.0001) among the groups. Female sex was predominant among all individuals evaluated and ranged from 55.5 to 63.8% (*p* = 0.35). 96.5% of NC were classified as MB and the most diagnosed clinical form was B (78.5%). Molecular diagnostic comparison (PCR-RLEP) showed 94.9% negative results for HHC and 43.7% positivity for *M. leprae* DNA in NC (*p* < 0.0001) (Table 1).

**Table 1:** Study population characteristics (*N* = 405).

	HEC *n* = 100	HHC *n* = 105	NC *n* = 200	*p*-value
Age, years, mean (SD)	58.5 (16.7)	41.1 (17.7)	54.2 (17.2)	**<0.0001**^**a**^
Sex, *n* (%)				
Male	40 (40.0)	38 (36.2)	89 (44.5)	0.35^b^
Female	60 (60.0)	67 (63.8)	111 (55.5)	
Operational Classification, *n* (%)				
PB	-	-	7 (3.5)	
MB	-	-	193 (96.5)	
Clinical Form, *n* (%)				
I	-	-	1 (0.5)	
TT	-	-	6 (3.0)	
B	-	-	157 (78.5)	
BL	-	-	5 (2.5)	
LL	-	-	10 (5.0)	
N	-	-	21 (10.5)	
PCR-RLEP, *n* (%)				
Negative	-	75 (94.9)^c^	80 (43.7)^d^	**<0.0001**^**b**^
Positive	-	3 (3.8)	61 (33.3)	

a Comparison by the Kruskal–Wallis test.

b Comparison by the Chi-square test.

c Data not available for 14 HHC.

d Data not available for 53 NC.

HEC, healthy endemic controls; HHC, household contacts of HD patients; NC, new cases of HD; SD, standard deviation; PB, paucibacillary; MB, multibacillary; I, indeterminate; TT, tuberculoid; B, borderline; BL, borderline lepromatous; LL, lepromatous; N, neural; PCR-RLEP, quantitative polymerase chain reaction-specific repetitive element.

### 4.2. Anti-Mce1A and anti-PGL-I antibodies are biomarkers for the diagnosis of patients and their contacts

**Figure 1:**
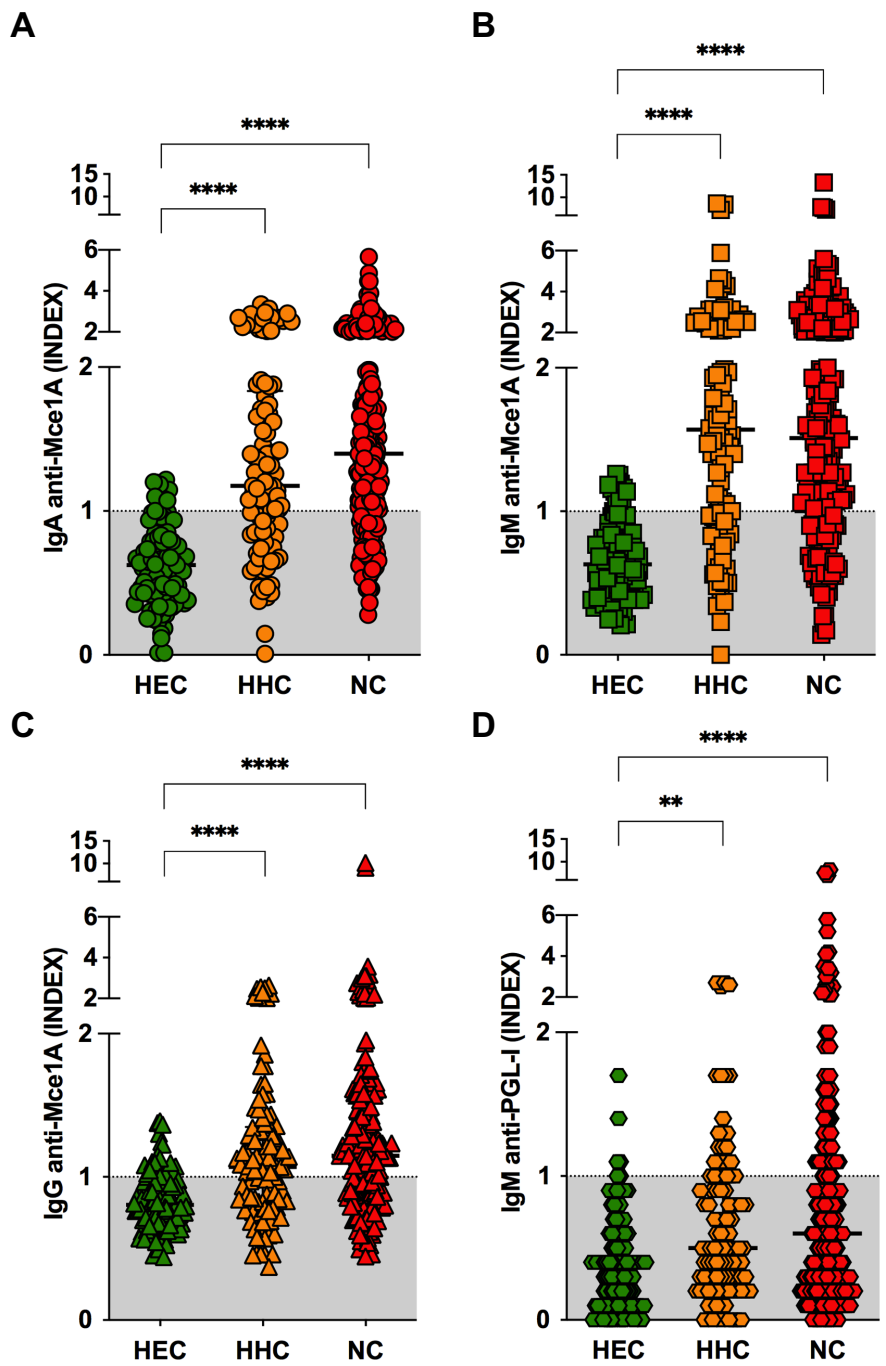
Biomarkers for the diagnosis of HD patients and their contacts. IgA **(A)**, IgM **(B)**, IgG **(C)** α-Mce1A, and anti-PGL-I **(D)** antibody indexes in plasma samples from HEC (*n* = 100), HHC (*n* = 105); and NC (*n* = 200). Statistical significance was determined by the Kruskal–Wallis test followed by the Dunn’s test; significance was considered at ^****^p < 0.0001 and ^**^p = 0.0041. The respective index was calculated by dividing the optical density (O.D. 450 nm) of each sample by the cut-off. Indexes above 1.0 were considered positive. HEC, healthy endemic controls; HHC, household contacts of HD patients; NC, new cases of HD; IgA, immunoglobulin A; IgM, immunoglobulin M; IgG, immunoglobulin G.

The antibody profiles of α-Mce1A protein and α-PGL-I indexes in newly diagnosed HD patients (NC), household contacts of HD patients (HHC), and healthy endemic-control individuals (HEC) are represented in Figure 1 as median and interquartile range (IQR). α-Mce1A IgA levels were significantly higher in the NC [median: 1.39 (IQR: 1.00–2.02), *p* < 0.0001] and HHC [median: 1.17 (IQR: 0.83–1.83), *p* < 0.0001] groups as compared to the HEC group [median: 0.62 (IQR: 0.42–0.81)] (Figure 1A). IgM α-Mce1A was evidently increased in HHC [median: 1.57 (IQR: 0.95–2.47), *p* < 0.0001] and NC [median: 1.51 (IQR: 1.025–2.32), *p* < 0.0001] as compared to HEC [median: 0.63 (IQR: 0.43–0.81)] (Figure 1B). α-Mce1A IgG indexes were higher in the NC [median: 1.14 (IQR: 0.87–1.51), *p* < 0.0001] and HHC [median: 1.070 (IQR: 0.80–1.34) *p* < 0.0001] groups than in HEC [median: 0.80 (IQR: 0.68–0.96)] (Figure 1C). The HHC group had moderate levels of α-PGL-I IgM [median: 0.50 (IQR: 0.30–1.0), *p* = 0.0041] as compared to HEC [median: 0.4 (IQR: 0.2–0.6)]. The NC indexes against PGL-I [median: 0.6 (IQR: 0.22–1.1)] showed significant differences compared to the HEC indexes (*p* < 0.0001) (Figure 1D).

### 4.3. Performance of anti-Mce1A antibodies and IgM anti-PGL-I for HD diagnosis

ROC curve analysis was performed to evaluate the performance of the three immunoglobulins against the Mce1A protein and IgM α-PGL-I for the diagnosis of NC, and the area under the curve (AUC), cut-off, sensitivity, and specificity values with 95% CI are shown in Table 2. α-Mce1A IgA had the best significant performance with AUC = 0.90 (CI: 0.87–0.93; *p* < 0.0001), with a case detection probability of 77.5% (CI: 71.1%–83.1%), and 89% (CI: 81.2%–94.4%) specificity. IgM showed a performance with AUC = 0.87 (CI: 0.83–0.91; *p* < 0.0001), with a 76.5% chance of correct classification (CI: 70.0%–82.2%) of new cases, and an 88% probability of identifying true negative individuals (CI: 80.0%–93.6%). The performance of the assay using IgA and IgM antibody was rated as having high accuracy (AUC > 0.85) for screening HD patients. The serological test with IgG showed AUC = 0.75 (CI: 0.69–0.80; *p* < 0.0001), 61.5% sensitivity (CI: 54.4%–68.3%) and 96% specificity (CI: 90.1–98.9) and was classified as having a moderate probability of providing correct results (AUC = 0.75–0.85). The α-PGL-I test showed performance with an AUC = 0.67 (CI: 0.61–0.72; *p* < 0.0001), 34.6% (CI: 28.5%–41.2%) probability of case detection, and 96% (CI: 90.1%–98.9%) specificity. The performance of α-PGL-I serology was classified as having low accuracy (AUC < 0.75). The absence of difference between NC and HHC in all analyses for immunoglobulin levels led to the evaluation of the ELISA performance only for the group of patients compared to controls (HEC).

**Table 2:** Comparison of the performance of IgA, IgM, and IgG α-Mce1A protein and IgM α-PGL-I for the diagnosis of new HD Cases (*n* = 200).

Antibody	AUC (95% CI)	*P*-value	Cut-off (O.D)	Sensitivity % (95% CI)	Specificity % (95% CI)	LR+
α-Mce1A IgA	0.90 (0.87–0.93)	<0.0001	0.189	77.5 (71.1–83.1)	89.0 (81.2–94.4)	7.045
α-Mce1A IgM	0.87 (0.83–0.91)	<0.0001	0.146	76.5 (70.0–82.2)	88.0 (80.0–93.6)	6.375
α-Mce1A IgG	0.74 (0.69–0.80)	<0.0001	0.172	61.5 (54.4–68.3)	79.0 (69.7–86.5)	2.929
α-PGL-I IgM	0.67 (0.61–0.72)	<0.0001	0.295	34.6 (28.5–41.2)	96.0 (90.1–98.9)	8.662

AUC, area under the curve; CI, confidence interval; O.D., optical density; LR+, positive likelihood ratio; Mce1A, mammalian cell-entry protein 1A; IgA, immunoglobulin A; IgM, immunoglobulin M; IgG, immunoglobulin G; PGL-I, phenolic glycolipid-I.

### 4.4. Positivity and evaluation of serological biomarkers in parallel

The performance of the α-Mce1A assay was also evaluated based on the percentages of biomarker seropositivity (Table 3). IgA α-Mce1A ELISA for NC was positive in 77.5% (155/200) of patients, IgM in 76.5% (153/200), IgG in 61.5% (123/200), and α-PGL-I serology in 28.0% (56/200) of positive NC. HHC were 11.8, 6.0, 4.5, and 8.0% less seropositive for the tested antibodies, respectively, as compared to NC. The use of the α-Mce1A immunoassay in NC compared with HEC showed 7.0x more positivity for IgA α-Mce1A, 6.4 for IgM and 2.9 for IgG. None of the assays performed with HEC samples showed antibody indexes ≥2.0. A positive serological test with a ≥ 2.0 index in HHC and NC, respectively, was obtained in 20.0 and 26.5% for IgA ELISA, in 32.4% and 34.0% for IgM ELISA, in 8.6% and 7.0% for IgG ELISA, and 3.8% and 2.0% for IgM α-PGL-I. The use of the new α-Mce1A IgA, IgM, and IgG biomarkers allowed an increase of 49.5%, 48.5%, and 33.5%, respectively, in the detection of NC as compared to the use of α-PGL-I serology.

**Table 3:** Positivity profile of serological biomarkers in HD diagnosis.

Seropositivity *n* (%)	HEC (*n* = 100)	HHC (*n* = 105)	NC (*n* = 200)
α-Mce1A IgA			
>1.0 < 2.0	11 (11.0)	48 (45.7)	102 (51.0)
≥2.0	0 (0)	21 (20)	53 (26.5)
Total	11 (11.0)	69 (65.7)	155 (77.5)
α-Mce1A IgM			
>1.0 < 2.0	12 (12.0)	40 (38.1)	85 (42.5)
≥ 2.0	0 (0)	34 (32.4)	68 (34.0)
Total	12 (12.0)	74 (70.5)	153 (76.5)
α-Mce1A IgG			
>1.0 < 2.0	21 (21.0)	51 (48.6)	109 (54.5)
≥ 2.0	0 (0)	9 (8.6)	14 (7.0)
Total	21 (21.0)	60 (57.2)	123 (61.5)
α-PGL-I IgM			
>1.0 < 2.0	4 (4.0)	17 (16.2)	39 (19.5)
≥ 2.0	0 (0)	4 (3.8)	17 (8.5)
Total	4 (4.0)	21(20.0)	56 (28.0)
α-Mce1A IgA + IgM + IgG			
>1.0 < 2.0	1 (1.0%)	10 (9.5)	26 (13.0)
≥ 2.0	0 (0)	5 (4.8)	8 (4.0)
Total	1 (1.0%)	15 (14.3)	34 (17.0)
α-Mce1A IgA + IgM			
>1.0 < 2.0	2 (2.0%)	15 (14.3)	44 (22.0)
≥ 2.0	0 (0)	6 (5.7)	16 (8.0)
Total	2 (2.0%)	21 (20.0)	60 (30.0)
α-Mce1A IgA + IgG			
>1.0 < 2.0	5 (5.0%)	1 (0.9)	61 (30.5)
≥ 2.0	0 (0)	8 (7.6)	11 (5.5)
Total	5 (5.0%)	9 (8.5)	72 (36.0)
α-Mce1A IgM + IgG			
>1.0 < 2.0	4 (4.0%)	23 (21.9)	48 (24.0)
≥ 2.0	0 (0)	6 (5.7)	10 (5.0)
Total	4 (4.0%)	29 (27.6)	58 (29.0)

HEC, healthy endemic controls; HHC, household contacts of HD patients; NC, new cases of HD; Mce1A, mammalian cell-entry protein 1A; IgA, immunoglobulin A; IgM, immunoglobulin M; IgG, immunoglobulin G.

Parallel analysis of markers with α-Mce1A ELISA showed results with up to 5.0% seropositivity for all antibodies tested in the HEC group and 14.3 and 17.0% for HHC and NC, respectively. Thus, the combination of positivity for two tested antibodies showed greater overlap for IgM + IgG in the HHC (27.6%) and for IgA + IgG in NC (36.0%), an increase of positivity of 5.7% for HHC and of 19.0% for NC, as compared to the serial evaluation with IgA + IgM + IgG. For all overlaps performed, NC showed better seropositivity results (Table 3).

The low seropositivity and accuracy of the α-PGL-I serology meant that the authors did not use it in the subsequent analyzes of the study.

### 4.5. Multivariate models employed to distinguish endemic controls, HD patients, and contacts by means of the New serological biomarkers

**Figure 2:**
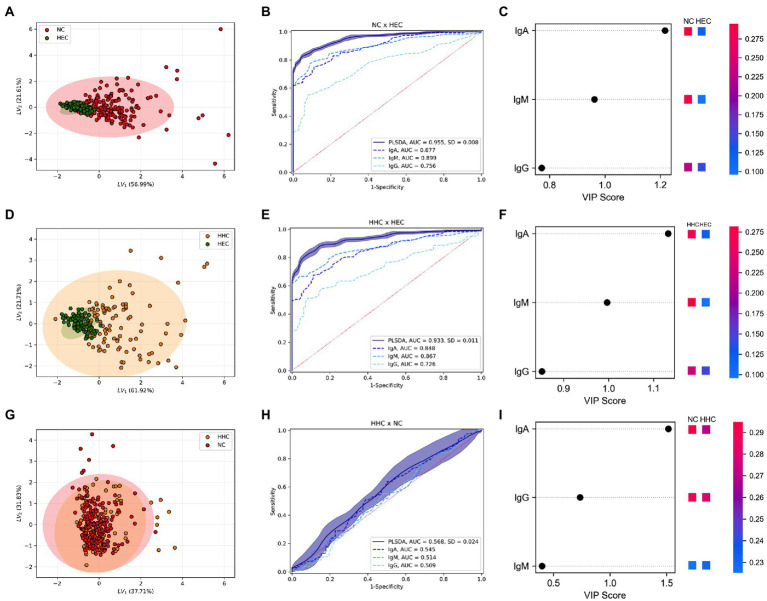
Simultaneous analysis of α-Mce1A antibodies in the clustering of groups. Partial least squares-discriminate analysis (PLS-DA) plot of IgA, IgM and IgG α-Mce1A combined from HEC, HHC, and NC. PLS-DA score scatter plots for HEC (green) and NC (red) **(A)**; HEC and HHC (yellow) **(D)**; HHC and NC **(G)**. Rank of the different immunoglobulins identified by PLS-DA according to the Variable Importance in Projection (VIP score) on the x-axis. The colored boxes on the right indicate the relative levels of the corresponding antibody (OD) in each group under study **(C,F**, and **I)**. Receiver operating characteristic (ROC) curve for schematic performance of PLS-DA classifiers over the validation set for combined antibodies and isolated levels in the NC and HEC **(B)**, HHC and HEC **(E)**, HHC and NC **(H)** groups. HEC, healthy endemic controls; HHC, household contacts of HD patients; NC, new cases of HD; IgA, immunoglobulin A; IgM, immunoglobulin M; IgG, immunoglobulin G.

The comparison of α-Mce1A antibody levels among NC, HHC, and HEC is shown in Figure 2. The performance of the model was evaluated using the intercept coefficient of determination (*R*^2^), predictive relevance (*Q*^2^), and significance of the permutation test (PT). Multivariate PLS-DA [*R*^2^ = 0.38 (SD:0.01); Q^2^ = 0.42 (SD:0.28); PT: *p* = 0.009] showed two defined clusters for the HEC and NC groups [accuracy = 0.95 (SD = 0.008)] and had the highest scores driving the cluster separation (LV1 = 56.99%) (Figure 2A). HEC and HHC [*R*^2^ = 0.40 (SD:0.01); *Q*^2^ = 0.36 (SD:0.32); PT: *p* = 0.009] also obtained excellent accuracy [accuracy = 0.93 (SD = 0.011)] and scores driving the cluster separation (LV1 = 61.92%) (Figure 2D). The analysis performance for the HHC and NC groups [*R*^2^ = 0.01 (SD:0.004); *Q*^2^ = -2.03 (SD:4.3); PT: *p* = 0.56] was not satisfactory [accuracy = 0.56 (SD = 0.024); LV1 = 37.7%] (Figure 2G). The ROC curve for model performance in discriminating the groups showed that IgM had the best accuracy in discriminating between HEC and NC (AUC = 0.87) (Figure 2B) and HEC and HHC (AUC = 0.86) (Figure 2E). The α-Mce1A IgG antibody showed the lowest accuracy among these groups (AUC = 0.75 and 0.72, respectively). α-Mce1A antibodies showed a low performance of IgA (AUC = 0.54), IgM (AUC = 0.51) and IgG (AUC = 0.50) in segregating HHC and NC due to the absence of difference in immunoglobulin levels in these groups (Figure 2H).

The ranking of the evaluated antibodies indicated that, in the discrimination among the groups after multivariate analysis, IgA α-Mce1A obtained a VIP score higher than 1 (VIP: 1.22; 1.13; 1.51), being the biomarker most responsible for the clustering of these groups (Figures 2C,F,I). The IgM antibody was the second relevant biomarker distinguishing between NC and HHC versus HEC (VIP: 0.96; 0.99, respectively). However, the IgG antibody was found to be the second most ideal biomarker only for the analyses between HHC and NC (VIP: 0.74) (Figure 2I).

### 4.6. Anti-Mce1A antibodies associated with HD diagnosis by means of Shapley values

**Figure 3:**
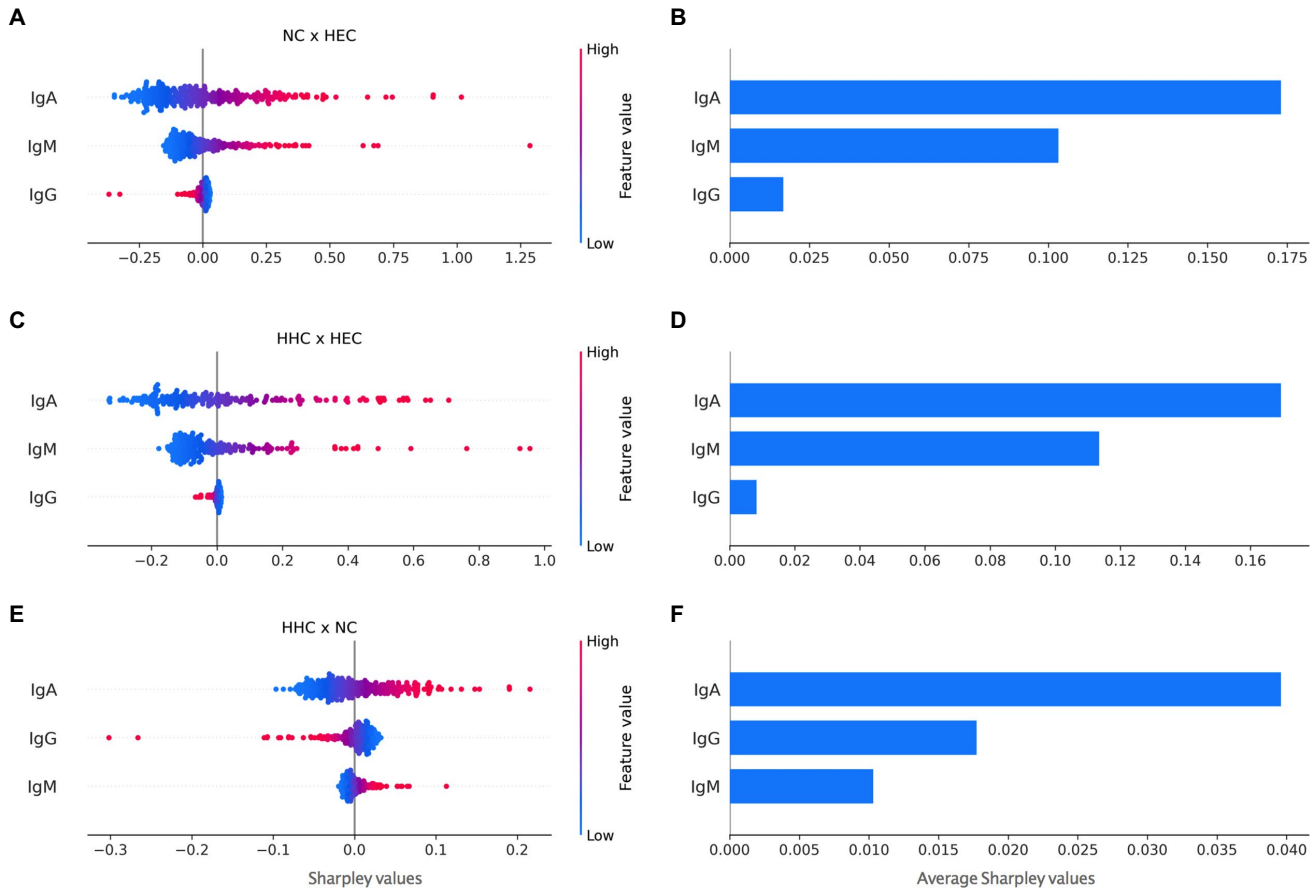
Contrasting Shapley values for impact on HD diagnosis for all antibodies against Mce1A protein. Each marker in the scatter plots corresponds to an individual and red to blue shades correspond to negatives to positive Shapley values **(A, C, E)**. The scatter plots expose not only the importance of a potential risk factor for HD diagnosis but also its range of effects over the NC and HEC **(A)**, HHC and HEC **(C)**, HHC and NC **(E)** groups. Scatter plots **(B,D**, and **F)** showed average Shapley values for the respective comparisons. HEC, healthy endemic controls; HHC, household contacts of HD patients; NC, new cases of HD; IgA, immunoglobulin A; IgM, immunoglobulin M; IgG, immunoglobulin G.

Comparative assessment of α-Mce1A antibody levels in HHC and NC had preferably positive Shapley values, suggesting that these conditions always tended to diagnose infection and/or disease. The values were represented as group means and as minimum and maximum values of individuals. Figures 3A,C,E plotted Shapley values for each individual while Figures 3B–F the average of the absolute values (modules). The IgA antibody showed the highest positive Shapley value in the analyses between HEC and NC (Figures 3A,B) i.e., 0.173 (range: −0.348-1.017), a value of 0.169 (range: −0.329-0.707) between HEC and HHC (Figures 3C,D), and a lower value of 0.039 (range: −0.096-0.215) between HHC and NC (Figures 3E,F). The Shapley values of IgG α-Mce1A for HHC and NC as compared to HHC and HEC appear clustered and partially negative [0.017 (−0.301–0.032)], thus suggesting that antibody positivity in these groups had less potential for association with the diagnosis of HD due to their similar response. On the other hand, the IgG antibody ranked better than the IgM α-Mce1A antibody in the evaluation of the difference between HHC and NC. IgM was found to be clustered and with most positive Shapley values (Figures 3E,F) [0.013 (−0.019–0.112)], thus being the marker that, after IgA, showed a positive impact on HD diagnosis between HHC and NC. In light of these results, the values obtained with the IgA and IgM α-Mce1A antibodies ranged from negative to positive for all group comparisons, thus suggesting that these conditions were always leaning toward HD diagnosis (Figures 3A–F).

Thus, the higher the IgA value, more PLS-DA tended to classify the individual as NC, and the lower its value or negative as HEC. The same is true for IgM. For IgG, the higher its value, the more the model tended to classify as HEC. This behavior was caused by the association of the IgG antibody with treated patients and low seropositivity in the diagnosis. Figure 3B shows that IgA contributed more than IgM, which contributed more than IgG. In Figure 3C, the higher the Shapley value, the more the model tended to classify as HHC. In Figure 3E, the higher the Shapley value, the more the model ranked the individual as NC.

### 4.7. Correlation of immunoglobulins against Mce1A protein

**Figure 4:**
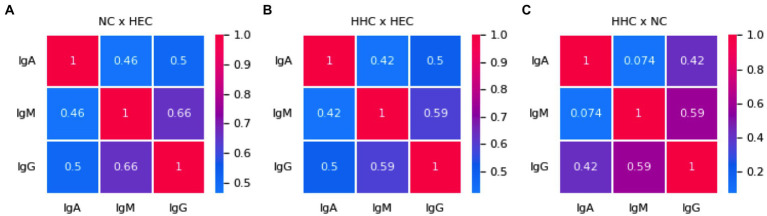
Immunoglobulins against Mce1A protein correlate weakly and moderately. Correlation matrix of α-Mce1A antibodies for NC and HEC **(A)**, HHC and HEC **(B)**, HHC and NC **(C)**. Spearman’s correlation coefficients between two pairs of variables are shown in the heatmap. Red to blue shades correspond to increasing values of Spearman’s correlation coefficient, as shown in the color bar. HEC, healthy endemic controls; HHC, household contacts of HD patients; NC, new cases of HD; IgA, immunoglobulin A; IgM, immunoglobulin M; IgG, immunoglobulin G.

Matrix correlation of α-Mce1A antibody levels among the study groups was calculated and the values are shown in color scale. NC and HEC showed a fair correlation between IgA and IgM (r = 0.46; *p* < 0.001) and between IgA and IgG (r = 0.50; *p* < 0.001). IgM and IgG showed a moderate positive correlation (r = 0.66; *p* < 0.001) between these two groups (Figure 4A). All positive correlations were fair for HHC and HEC, with *r* = 0.42–0.59 (*p* < 0.001) (Figure 4B). The correlation between HHC and NC for IgA and IgM was poor (*r* = 0.074; *p* = 0.186) and the correlation for IgA versus IgG and for IgM versus IgG was classified as fair (r = 0.42; *p* < 0.001) and moderate (*r* = 0.59; *p* < 0.001), respectively (Figure 4C). Further analyses demonstrated that α-Mce1A IgA correlated poor (*r* = 0.15; *p* = 0.04), IgM, and IgG (*r* = 0.37; *p* < 0.0001) fair with α-PGL-I indices. The proposed assay with Mce1A was able to detect different individuals in comparison with PGL-I serology.

### 4.8. Anti-Mce1A serology was able to provide hierarchical clustering for the individuals evaluated

**Figure 5:**
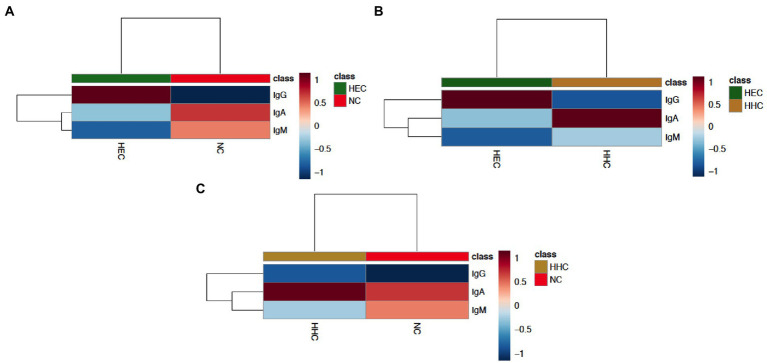
The indexes of anti-Mce1A biomarkers can cluster hierarchically. The Clustering result is shown as a dendrogram and heatmap (distance measure using similarity measure - Euclidean distance, and algorithm using clustering to minimize the sum of squares of any two clusters - Ward’s linkage). Hierarchical cluster analysis was performed using normalized and transformed antibody indexes. Each sample begins as a separate cluster and the algorithm proceeds to combine them until all samples belong to one cluster for HEC and NC **(A)**, HEC and HHC **(B)**, and HHC and NC **(C)**. The heatmap shows the dendrogram data values transformed into an average color scale with high values in red and low values in blue. HEC, healthy endemic controls; HHC, household contacts of HD patients; NC, new cases of HD; IgA, immunoglobulin A; IgM, immunoglobulin M; IgG, immunoglobulin G.

We combined these plasma antibodies indexes with the group’s classification in HEC, HHC, and NC to apply machine learning using hierarchical methods of cluster analysis and as the main objective of the algorithm to provide the level of importance of each biomarker for each group through the heatmap. The following results were obtained: α-Mce1A IgA and IgM serology yielded essential results for NC identification as compared to HEC (Figure 5A); positivity for ELISA IgA was responsible for the clustering of HHC, while IgG ELISA was responsible for the clustering of HEC (Figure 5B), showing a very low involvement of IgM serology in the clustering of these two groups (HHC and HEC); positive samples for IgA and IgM distinguished NC from HHC, with IgA being the most intense antibody in terms of clustering performance in the HHC group (Figure 5C).

## 5. Discussion

**Figure 6:**
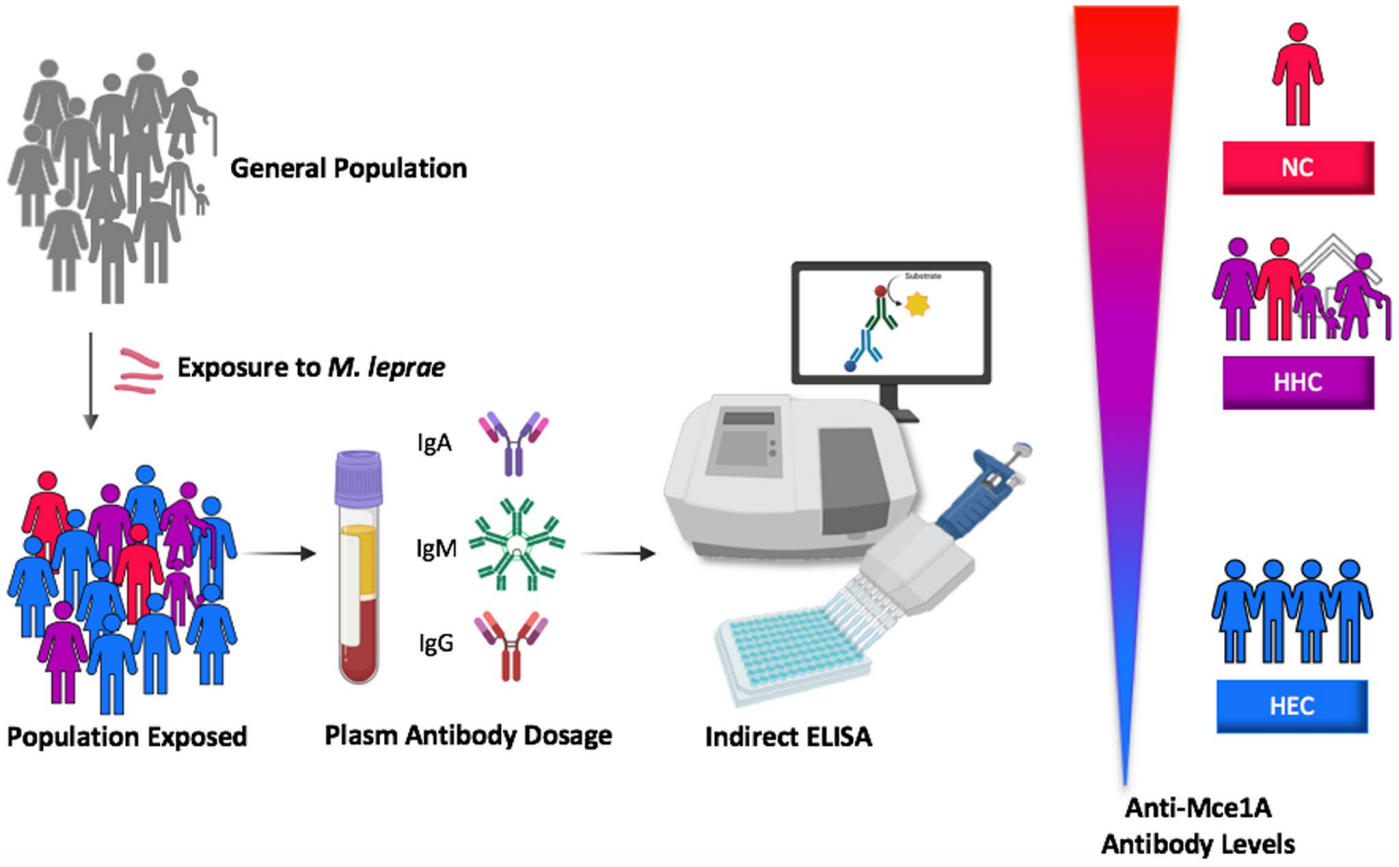
Proposed model of the stimulus and utility of specific antibodies against Mce1A protein in the laboratory diagnosis of HD based on the level of seropositivity for patients, household contacts and endemic controls. HEC, healthy endemic controls; HHC, household contacts of HD patients; NC, new cases of HD; Mce1A, mammalian cell-entry protein 1A.

The present results confirm the biomarker potential of α-Mce1A antibodies in the diagnosis of patients with HD, the screening of their contacts, and the assessment of exposure to the bacillus in endemic regions (Figure 6). The published results (11, 12) of the analysis with antibody levels in the different clinical forms and operational classification do not show differences between these groups for levels of α-Mce1A immunoglobulins. Also, there is no correlation or association between PCR positivity and bacillary load with positivity or higher levels of α-Mce1A antibodies in the tested samples. Thus, α-Mce1A serology differs from the α-PGL-I tool, which has been consolidated in the literature for correlation with bacillary load, operational classification, and multibacillary clinical forms. Therefore, the work analysis strategies aimed to identify patients with HD regardless of clinical classification and laboratory results for PCR, bacilloscopy, and α-PGL-I serology.

Serological testing for IgA is presented as an additional tool for the diagnosis and classification of HD, with potential utility for exposure monitoring of household contacts. In agreement with our data, Silva et al. (19) reported greater IgA reactivity against the conjugated antigen formed by natural octyl disaccharide linked to human serum albumin (NDO-HSA) among household contacts of PB and MB patients than among non-endemic controls. Accordingly, α-Mce1A IgA showed satisfactory accuracy (AUC 0.90) with 77.5% sensitivity and 89.0% specificity and revealed greater seropositivity of the immunoglobulins tested in patients, with 77.5% for new cases and 65.7% for contacts. In parallel, our analyses show that IgA was the antibody most responsible for clustering contacts between patients and endemic controls.

IgA is an antibody associated with the mucosal response, the main gateway of the bacillus in the establishment of infection, participating in the early stages of HD and in subclinical infection (19–21). The importance of IgA for host mucosal immunity is well established and, although its role in the systemic circulation has not been fully elucidated (22), its usefulness as an immunological marker in laboratory tests has been confirmed.

Most published studies use IgM as a target molecule in serological assays in view of the fact that the seroprevalence of α-PGL-I IgM is higher than the seroprevalence of IgA and IgG in endemic areas (23). IgM-seropositive individuals are at higher risk of developing the disease (24); however, IgM seropositivity is not predictive of the disease, as demonstrated with α-PGL-I IgG (5, 25). The findings using IgM and α-PGL-I IgG corroborate the data obtained with α-Mce1A serology. In the evaluation of previous *M. leprae* infection as a risk factor for diagnosed transmission through IgM α-PGL-I serology, IgM represents a biomarker of greater sensitivity than IgG since it can be detected in many individuals already infected with the bacillus despite the absence of disease (23, 26, 27). The diagnostic performance of α-PGL-I ELISA was only 28.0% for new cases of HD and 20.0% for their contacts, with a 34.6% probability of case detection, and 96.0% specificity. Thus, 48.5% fewer positives were identified in comparison with IgM α-Mce1A serology.

IgM α-Mce1A had high accuracy (AUC 0.87) with a chance of correct classification of 76.5% of new cases and 88.0% specificity; 76.5% of newly diagnosed patients with the disease and 70.5% of household contacts were seropositive in ELISA.

Positive results with a high index indicate the priority for screening, new clinical and laboratory evaluations, and monitoring of contacts, mainly with indexes ≥2.0. These results were obtained here in 32.4% of contacts and 34.0% of new HD cases with positivity in the IgM α-Mce1A immunoassay. Thus, IgM is the key antibody for the clustering of new cases in relation to the other groups evaluated (HHC and HEC).

The ELISA results for all tested immunoglobulins were negative for an index ≥2.0 in healthy individuals from the endemic region, emphasizing the importance of serologies with a high value of seropositivity (≥ 2.0) in patients and their contacts. IgA serology with values ≥2.0 was positive in 26.5% of patients (NC) and 20.0% of contacts. On the other hand, rates higher than ≥2.0 for IgG α-Mce1A were only detected in 7.0% of the cases and 8.6% of the contacts. Thus, having positive serology for the contact and the case demonstrates the need for greater clinical surveillance of these individuals and the differentiation between these groups will be based on the clinical diagnosis, which remains the confirmatory evidence and gold standard for the diagnosis of HD.

Disease control and protective immunity in HD are associated with effective cellular immunity of T-cell responses. Studies evaluating antibody responses are primarily focused on their utility as a serological diagnostic tool. Rada et al. (28) showed that IgG responses decrease in MB and PB patients during treatment with MDT when using IgG against *M. leprae* antigens such as ML0405, ML2331, and LID-1 aiming to monitor the treatment of patients with the non-reactive LL form. However, data from assays targeting the detection of α-Mce1A IgG show variable seropositivity according to endemicity, showing that there may be a lower frequency of positives (61.5%) in less endemic regions and a high seroprevalence in new cases in a hyperendemic region (84.0%), as reported by Lima et al. (12). Patients treated with MDT had the highest rate of IgG positivity, which was detected in 89.5% of the patients evaluated in the study.

Recently, different cases of patients treated at an emergency unit in the Brazilian southeast were clinically diagnosed with HD and characterized by presenting hypoanesthetic skin lesions and thickened nerves, with peripheral nerve ultrasound demonstrating asymmetric and focal multiple mononeuropathy, and also with a positive molecular diagnosis in all patients tested by RLEP-PCR. Confirming the potential and innovative aspect of the new markers proposed for HD serology, 71.4, 100, and 42.8% of patients were positive for IgA, IgM, and IgG α-Mce1A, respectively. However, 100% were negative for α-PGL-I IgM (29).

Laboratory assays using α-PGL-I and α-LID-1 by ELISA and rapid test platforms with NDO-LID show low sensitivity and accuracy and are not recommended for isolated use in the diagnosis of HD, considering the complexity of the immunological presentations and the clinical aspects of the disease. A study by Frade et al. (30) demonstrated 48%–62% sensitivity and 70% specificity for α-PGL-I and α-LID ELISA and 40% specificity for NDO-LID. Other reports evaluating different studies with protocols using the PGL-I antigen demonstrated an average sensitivity of 63.8% and an average specificity of 91% as a diagnostic method in HD but are indicated mainly in MB cases, due to low positivity in PB cases (6).

The development of serological tests using antigens shared by a genus of a pathogen requires the evaluation of potential factors that can cause cross-reactivity in the results, such as vaccination with BCG, which is widespread in Brazil (> 90%) (31). The response to α-Mce1A antibodies was evaluated by Lima et al. (2017) in individuals with one or two BCG scars. However, in this study, we did not evaluate the response in newly vaccinated contacts and the proposal of the serological diagnosis during the clinical investigation of the patients and contacts before any prophylactic and/or therapeutic method for HD. Levels of antibodies against *M. tuberculosis* proteins (32) and LTBI did not induce distinct levels of α-Mce1A antibodies in the diagnosis of HD patients (11, 12, 33). A linear immunodominant epitope KRRITPKD (residues 131 and 138 in Mce1A) is highly conserved in *M. tuberculosis*, which is a possible explanation for the difference in response between patients with tuberculosis and HD, despite the homology between the mce1 gene (12, 34). Thus, allowing less chance of cross-reactions between individuals infected with both species of mycobacteria. However, it is a limitation of the α-Mce1A antibody assay for diagnosing HD in patients also diagnosed with or with a recent history of active tuberculosis. We sought to use three different antibodies (IgA, IgM, and IgG) to minimize bias and ensure the different proposed interpretations, such as diagnosis, potential subclinical infection, contact with *M. leprae*, and patients already treated for HD.

In parallel, for the determination of the cut-off value in populations with different endemic profiles, we need to know the pretest probability of the disease of interest as well as the costs incurred by misdiagnosis. Accordingly, the cut-off value is not universal and should be determined for each region and each disease condition according to endemicity (35). We still do not have commercially available serological tests capable of detecting cases with high sensitivity and accuracy. Thus, more exploratory studies to characterize new molecules capable of providing an immunological signature with high sensitivity and maintaining specificity are implemented as an advance in the search for new technologies to aid in the diagnosis of HD and the screening of contacts. Currently, α-Mce1A serology has not been able to distinguish contacts and patients with active disease, requiring further studies to understand whether seropositivity for the markers among household contacts is a predictor of the development of active disease or will only allow the identification of the contact with the bacillus regardless of disease progression. The seropositivity pattern of contacts for the tested immunoglobulins, similar to that found in patients and absent in healthy endemic controls, contributes as one more alert test for better clinical follow-up of positive contacts. α-Mce1A serology corrects the main shortcomings in accuracy of the previous serology (PGL-I), as it demonstrates greater sensitivity, regardless of the clinical form or bacillary load.

The PCR-RLEP technique proved to be a methodology for identifying patients at diagnosis due to its positive rate (33.3%). The molecular technique showed performance with a positivity rate of 3.8% in household contacts. Thus, it represents another high-specificity diagnostic platform assisting with the diagnosis and screening of potential subclinical cases. Sensitivity can range from 51% to 91%, and specificity from 46% to 100% (6). A study published in the same endemic region identified a PCR positivity rate of 41.0% and sensitivity and specificity of 41.0 and 100% for HD patients, respectively (7). The evaluation of cases using complex neurological assessment techniques permits a better classification of patients into MB forms (2). In the present study more than 70.0% of the cases were diagnosed with the B clinical form and 96.5% with the MB form, mainly in patients with atypical hypochromatic macules with altered sensation, neurological findings on hands and feet, and lower bacillary load.

In line with the search for new tools for an early diagnosis such as ELISA α-Mce1A, the treatment of these cases is the next step toward achieving the goal of eliminating the disease in the community. As reported by the WHO, case detection and treatment with MDT alone are insufficient strategies to interrupt transmission. Thus, to boost the prevention of HD, the current recommendation is an active search of the household and social contacts of each patient, accompanied by the offer of preventive chemotherapy (3).

In summary, the present data suggest that combined serological testing based on IgA, IgM, and IgG α-Mce1A antibodies should be performed in order to ensure an interpretation of the three possibilities proposed for the new markers: positive IgA and/or IgG indicative of contact with the bacillus due to the strong positive correlation between these antibodies; positive IgM for diagnosis or priority for further clinical follow-up of contacts; Negative IgM and positive IgG as a form of therapeutic monitoring after MDT use. Serological assays are complementary diagnostic platforms, clinical correlation is always necessary and the region’s endemicity is considered. Finally, the incorporation of new diagnostic technologies makes it possible to eliminate the main gaps in the laboratory diagnosis of HD with the implementation of tools of greater sensitivity and accuracy while maintaining satisfactory specificity. This procedure contributes to the goals of the WHO for the identification of initial and infected cases and for the interruption of bacillary transmission in the family environment, effectively reaching zero disability and eliminating the stigma of Hansen’s disease.

## Data availability statement

The original contributions presented in the study are included in the article/Supplementary material, further inquiries can be directed to the corresponding author.

## Ethics statement

The studies involving human participants were reviewed and approved by Institutional Review Board for Human Research of HCFMRP-USP (Protocol number 4.142.534/2020). The patients/participants provided their written informed consent to participate in this study.

## Author contributions

FL and MF substantially contributed to study conception and design, acquisition of data, and/or analysis and interpretation of data. HL, FP, NF, and MF contributed to the clinical care of patients. VA, GF, and GD provided acquisition of clinical data. FL and NP executed and interpreted the laboratory tests. FL, MS, and DT contributed to statistical analysis and interpretation of the data. LR and SA provided scientific guidance and advice. MF gave final approval of the final submitted version and provided supervision and orientation of the study. All authors contributed to the interpretation of the results and to critical revision.

## Funding

This study was financed in part by Coordenação de Aperfeiçoamento de Pessoal de Nível Superior-Brazil (CAPES)-Finance Code 001 and with Ph.D. scholarships for GM and MS, by the National Council for Scientific and Technological Development (CNPq) with Ph.D. scholarships for FL and a research grant for MF (423635/2018–2), by the Unified Scholarships Program (PUB-USP) for VA and GF, by Fundação de Amparo à Pesquisa do Estado de São Paulo (FAPESP) with a research grant 2021/13429–1, by the Brazilian Health Ministry (MS/FAEPA-FMRP-USP: 749145/2010 and 767202/2011), and by the Oswaldo Cruz Foundation-Ribeirão Preto (TED 163/2019–Protocol N° 25380.102201/2019-62/Project Fiotec: PRES-009-FIO-20). We also acknowledge the financial support of the Research and Assistance Support Foundation of the Hospital of the Medical School of Ribeirão Preto at USP (FAEPA) to the National Referral Center for Sanitary Dermatology and HD.

## Conflict of interest

The authors declare that the research was conducted in the absence of any commercial or financial relationships that could be construed as a potential conflict of interest.

## Publisher’s note

All claims expressed in this article are solely those of the authors and do not necessarily represent those of their affiliated organizations, or those of the publisher, the editors and the reviewers. Any product that may be evaluated in this article, or claim that may be made by its manufacturer, is not guaranteed or endorsed by the publisher.
